# Book Notices

**Published:** 1882-08-20

**Authors:** 


### *BOOK NOTICES.*

DISEASES OF THE RECTUM AND ANNUS. By Charles B. Kelsey, M. D., Surgeon to St. Paul's Infirmary for Diseases of the Rectum, etc.; etc. New York: Wm. Wood & Co.

This is an illustrated work of 288 oc. pages, well gotten up and contains a great amount of valuable, practical information upon a department but little understood by the profession at large. It is well adapted to both the student and the general practitioner.

ANNUAL REPORT of the Board of Regents of the Smithsonian Institute, showing the operations, expenditures and condition of the Institution for the year 1880. Washington: Government Printing Office, 1881.

The work exhibits the financial affairs of the Institution, etc. The Appendix contains a record of recent progress in the principal departments of science; astronomical observations, etc., for the past year from the various Observatories in the United States are given.

PRINCIPLES OF HUMAN PHYSIOLOGY. By Wm. B. Carpenter, M. D., F. R. S., F. G. S., F. L. S., Registrar to the University of London, etc. Edited by Henry Power, M. B., F. R. C. S., London, Examiner in Natural Science, etc., etc. A new American from the Eighth revised and enlarged English edition, with notes and additions by Frances G. Smith, M. D., Professor of Institues of Medicine in the University of Pennsylvania, etc. Philadelphia: Henry C. Lea, 1876.

We can recommend the above as one of the best works extant on Physiology. Suited to both the general practitioner and to the student of medicine.

ELECTRICITY IN SUGERY. By John Butler, M. D. Boericke & Tafel, New York, 145 Grand street. Philadelphia, 1011 Arch street, 1882.

A work of 109 pages. Practical, and based upon the author's experience, and designed for the use of the specialist and general practitioner.

FOURTH BIENNIAL REPORT of the State Board of Health of Maryland, 1882.

We are indebted to Dr. C. W. Chancellor, Secretary, for a copy of the above interesting Report. The following are the officers of the Board, to-wit:

J. Robert Ward, M. D., President, Govanstown.
C. W. Chancellor, M. D., Secretary, Baltimore.
James A. Stewart, M. D., Baltimore.
Hon. Charles J. M. Gwinn, Baltimore.
J. Crawford Nelson, C. E., Baltimore.
St. George W. Teackle, M. D., Baltimore.

TRANSACTIONS of the American Gynecological Society, Vol. 6, for the year 1881. Philadelphia: Henry C. Lea's Son & Co.

This volume contains the index to the Gynecological and Obstetric literature of all countries, for the year 1880, prepared with the co-operation of Dr. J. S. Billings, U. S. A., in charge of the National Medical Library, Washington.

MENTAL PATHOLOGY AND THERAPEUOICS. By W. Griesinger, M. D., Professor of Clinical Medicine and of Mental Science in the University of Berlin, Homorary member of the Medico-Psychological Association, membre Associe Etranger De La Societe Medico-Psycologique De Paris, etc., etc. Translated from the German (Second edition) by C. Lockhart Robertson, M. D., Cantab, Medical Superintendent of the Sussex Lunatic Asylum, and James Rutcherford, M. D.. Edin.

A work of 361 oc. pages, in which are treated with much ability the various forms of insanity, melancholia, mania, dementia, and other complications. Interesting and important information on psycology, idiocy, cretinism, etc., are given, and other matters pertaining to mental disorders are treated in a manner at once forcible and interesting.

MULTUM IN PARVO—the best Pocket Anatomist Founded upon Gray—By C. Henri Leonard, A. M., M. D., Professor of the Medical and Surgical Diseases of Women and Clinical Gynecology in Michigan Medical College, etc.

Eleventh revised edition. A useful little work for students at the dissecting table. Price, 75 cents. C. H. Leonard, 89 Miami Avenue, Detroit, Mich.

THE INCIDENTAL EFFECTS OF DRUGS: A Pharmacological and Clinical Hand Book, by Dr. L. Lewin, Assistant at the Pharmaceutical Institute of the University of Berlin. Translated by W. T. Alexander, M. D. New York: William Wood & Co., 1882. McGarity & Laird, Agents, Atlanta, Ga. Octavo, 239 pages.

This work is one of unusual interest, and should be carefully read by the practitioner, who will here find a solution of many conditions and symptoms not heretofore understood, and supposed to be different phases of disease, when in reality they are the result of the incidental effects of the medicines used. In the case of quinine, for instance, we sometimes see "headache, deafness, general muscular excitability, chilliness and vertigo disappearing in the horizontal position. Sometimes periodical anxiety, fainting or symptoms of collapse." A species of urticaria is also not unfrequently seen, and other forms of eruption. It will be a useful and interesting work of reference, and should be in the library of every practitioner.

TRANSACTIONS of the Medical and Chirurgical Faculty of the State of Maryland, at its eighty-fourth Annual Session held at Baltimore.

The volume is a neat one, and contains many able and interesting papers, of which we have not space to make special mention. The President elect for the present year is Dr. Wm. M. Kemp.

Vice-Presidents: Dr. Tho. S. Latimer and Dr. Richard McSperry.

Recording Secretary: W. L. Register, M. D.

WHAT TO DO IN CASES OF POISONING. By William Murrell, M. D., M. R. C. P., Lecturer on Materia Medica and Therapeutics at Westminster Hospital, Assistant Physician to the Royal Hospital for Diseases of the Chest. Second edition. Detroit, Mich., U. S. A., 1882. Geo. S. Davis, publisher.

A little work of much useful and practical information.

